# AskBeacon—performing genomic data exchange and analytics with natural language

**DOI:** 10.1093/bioinformatics/btaf079

**Published:** 2025-02-22

**Authors:** Anuradha Wickramarachchi, Shakila Tonni, Sonali Majumdar, Sarvnaz Karimi, Sulev Kõks, Brendan Hosking, Jordi Rambla, Natalie A Twine, Yatish Jain, Denis C Bauer

**Affiliations:** Australian e-Health Research Centre, Commonwealth Scientific and Industrial Research Organisation, Adelaide, SA 5000, Australia; Data61, Commonwealth Scientific and Industrial Research Organisation, Sydney, NSW 2015, Australia; Data61, Commonwealth Scientific and Industrial Research Organisation, Sydney, NSW 2015, Australia; Data61, Commonwealth Scientific and Industrial Research Organisation, Sydney, NSW 2015, Australia; Centre for Molecular Medicine and Innovative Therapeutics, Murdoch University, Perth, WA 6150, Australia; Perron Institute for Neurological and Translational Science, Perth, WA 6009, Australia; Australian e-Health Research Centre, Commonwealth Scientific and Industrial Research Organisation, Sydney, NSW 2145, Australia; Centre for Genomic Regulation (CRG), The Barcelona Institute of Science and Technology, Barcelona, Ciutat Vella 08003, Spain; Department of Medicine and Life Sciences, Universitat Pompeu Fabra, PRBB, Barcelona 08003, Spain; Australian e-Health Research Centre, Commonwealth Scientific and Industrial Research Organisation, Sydney, NSW 2145, Australia; Faculty of Science and Engineering, Applied BioSciences, Macquarie University, Macquarie Park, NSW 2109, Australia; Australian e-Health Research Centre, Commonwealth Scientific and Industrial Research Organisation, Sydney, NSW 2145, Australia; Faculty of Science and Engineering, Applied BioSciences, Macquarie University, Macquarie Park, NSW 2109, Australia; Australian e-Health Research Centre, Commonwealth Scientific and Industrial Research Organisation, Adelaide, SA 5000, Australia; Faculty of Science and Engineering, Applied BioSciences, Macquarie University, Macquarie Park, NSW 2109, Australia; Department of Biomedical Informatics and Digital Health, School of Medical Sciences, University of Sydney, Sydney, NSW 2006, Australia

## Abstract

**Motivation:**

Enabling clinicians and researchers to directly interact with global genomic data resources by removing technological barriers is vital for medical genomics. AskBeacon enables large language models (LLMs) to be applied to securely shared cohorts via the Global Alliance for Genomics and Health Beacon protocol. By simply “asking” Beacon, actionable insights can be gained, analyzed, and made publication-ready.

**Results:**

In the Parkinson's Progression Markers Initiative (PPMI), we use natural language to ask whether the sex-differences observed in Parkinson's disease are due to X-linked or autosomal markers. AskBeacon returns a publication-ready visualization showing that for PPMI the autosomal marker occurred 1.4 times more often in males with Parkinson’s disease than females, compared to no differences for the X-linked marker. We evaluate commercial and open-weight LLM models, as well as different architectures to identify the best strategy for translating research questions to Beacon queries. AskBeacon implements extensive safety guardrails to ensure that genomic data is not exposed to the LLM directly, and that generated code for data extraction, analysis and visualization process is sanitized and hallucination resistant, so data cannot be leaked or falsified.

**Availability and implementation:**

AskBeacon is available at https://github.com/aehrc/AskBeacon.

## Graphical abstract

**Graphical abstract:**
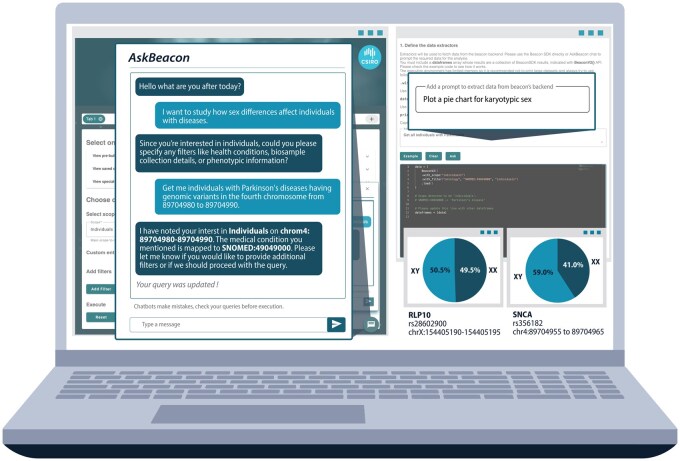

## 1 Introduction

Global Alliance for Genomics and Health (GA4GH) introduced the Beacon protocol (Rueda *et al.* 2022) to standardize the exchange of genomic and phenotypic information. The underlying schema is designed for clinical and research use and enables industry-standard security and data governance practices. Beacon can jointly query genotypic and phenotypic data, with metadata information encoded through ontologies. However, empowering the community to perform these advanced queries requires a user-interface that can hide the underlying complexities, such as query combinations across collections (Cohorts and Datasets), entity types (Individuals, Biosamples, Runs, Analyses and Genomic variants), and return types (Records, Boolean, Counts), as well as translating medical or colloquial terminology to an ontology code, e.g. SNOMED.

**Figure 1.**
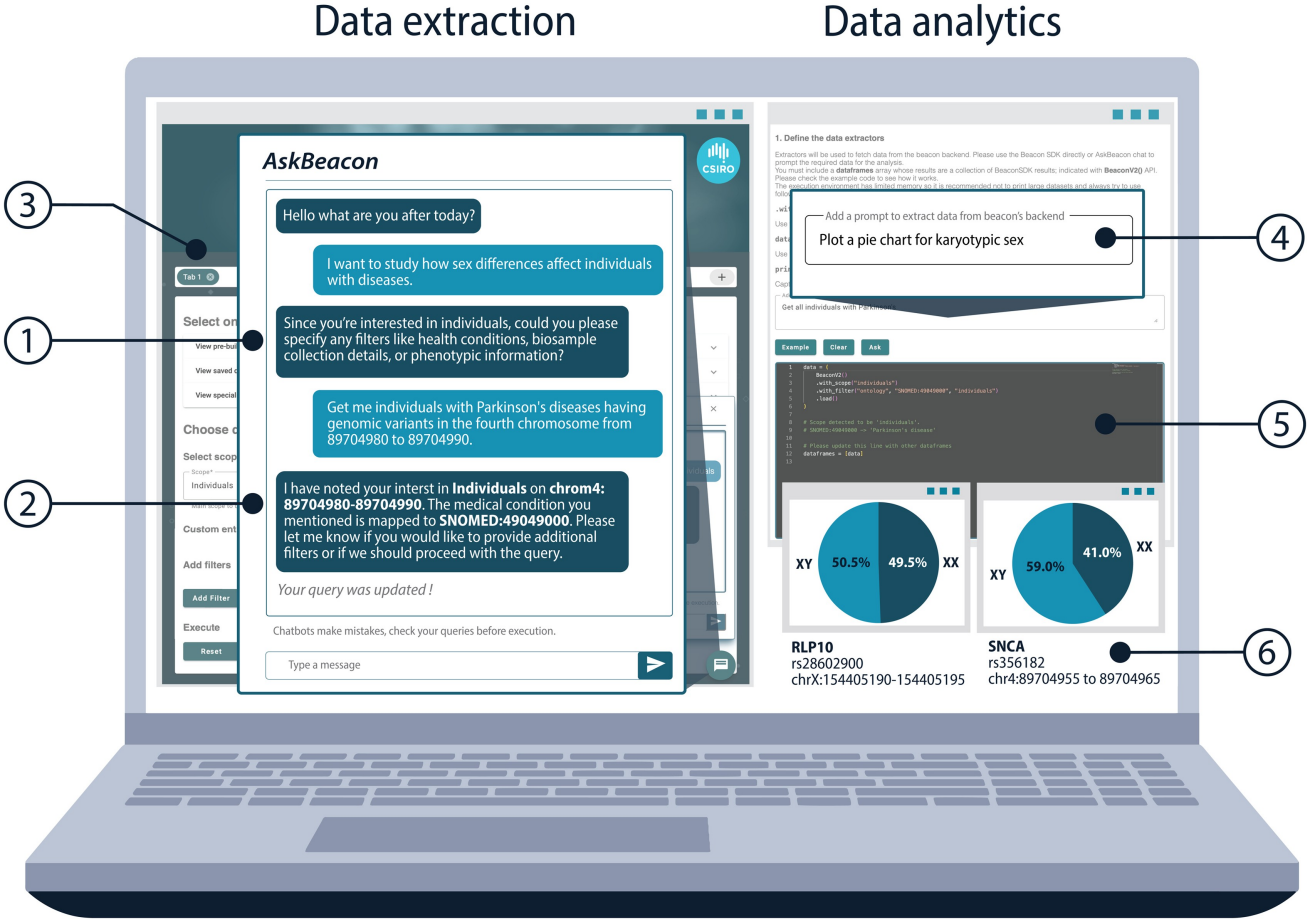
Gaining actionable insights on genomic data. (1) AskBeacon works with the user to create a valid beacon query. (2) Checks with the User that all inferred information is correct (e.g. ontology term, locations, etc.). (3) The user interface has different tabs to process queries in parallel and keeping contexts separate. (4) AskBeacon performs data analytics from prompts, presenting the resulting code to the user for optional verification and refinement (5). It creates publication-ready visualizations (6).

We developed AskBeacon to abstract the complexities of the Beacon schema using large language models (LLMs). AskBeacon is a web interface (Supplementary Section S1) on top of sBeacon (Wickramarachchi *et al.* 2023), a cloud-based production-ready implementation of the Beacon protocol. It acts as the interpreter between a clinical or research question and the Beacon schema-formatted query (Fig. 1).

For example, using AskBeacon, clinicians and researchers can validate in their data whether the genetically determined sex differences in Parkinson's Disease (Klein and König 2021) are due to X-linked [*RPL10* (Le Guen *et al.* 2021)] or autosomal [*SNCA* (Angelini *et al.* 2024)] genetic factors. Using natural language, users can perform currently expert-only steps such as (i) translating “Parkinson’s disease” to an ontology code, (ii) identifying individuals in the cohort with the relevant genotypes, (iii) constructing the right query to obtain the data, and (iv) analysing the data using custom scripts to visualize the results.

AskBeacon can query across federated repositories (Sever 2023) in the global Beacon Network (Fiume *et al.* 2019), where each node can be stood up securely and efficiently using an implementation of the Beacon protocol (Fromont *et al.* 2024), like sBeacon. This enables even small clinical and research groups, e.g. from underrepresented populations, to share genomic data and enable the secure, fully consented, and controlled query across human genetic diversity (Supplementary Section S2).

With our examples (Supplementary Section S6.3), we aim to inspire end-users like clinicians and researchers, and by addressing the paper to data providers we hope to empower them to meet the demand for interactivity by standing up an askBeacon-augmented sBeacon (Wickramarachchi *et al.* 2023) instance.

## 2 Methods

AskBeacon implements LLM connectors for the Beacon schema. AskBeacon is methodology agnostic, enabling new LLMs to be added as they become available. We tested the chat facility of currently available open [Ollama (2024), HuggingFace (Vaswani *et al.* 2017)] and commercial or closed-weighted models [OpenAI models GPT3.5 (Ye *et al.* 2023) and GPT 4 (Achiam *et al.* 2023), Anthropic model Claude 3.5 (Anthropic 2024)], as well as two different architectures (parallel and multi-step) for extracting the necessary information to populate a valid Beacon query (Supplementary Section S3). Specifically, we tested the individual components of a successful query, such as scope extraction, granularity extraction, variants extraction, and filter extraction as well as query validity (Supplementary Section S4).

## 3 Results

### 3.1 AskBeacon joins convenience of LLM with security of Beacon

For the research question “I want to study how sex differences affect individuals with disease,” AskBeacon guides the user with prompts until all information for a successful Beacon query is presented (Fig. 1.1). AskBeacon does the heavy lifting of obtaining ontology terms, interpreting genomic locations, inferring the query structure, and confirming all of this with the user (Fig. 1.2). The actual query is executed by Beacon’s software development kit (SDK, Supplementary Section S6), guaranteeing deterministic results. In our example, the data extraction obtains Individuals with mutations in rs28602900 (*RPL10*) and rs356181 (*SNCA*) from the PPMI (Chahine *et al.* 2018) (http://www.ppmi-info.org see acknowledgement). For beacon instance that are authorized to provide numeric return values, AskBeacon also supports data analytics with prompts such as “Plot a pie chart for karyotypic sex” (Fig. 1.4). It automatically generates the required analysis code and displays it for the user to confirm or adjust (Fig. 1.5). In our case, Fig. 1.6 shows that the X-linked marker (*RPL10*) has no sex difference in PPMI, while the autosomal marker on chromosome 4 occurs 1.4 times more often in males with Parkinson’s than females.

### 3.2 Modular use of LLM to keep pace with innovation

The parallel extraction approach was more resilient to network failures, incorrect inputs, partial inputs, and malformed LLM, however, it consumes more tokens compared to multi-step extractor. The multi-step approach can instead pick the suitable next chain depending on previous input or terminate chains early resulting in lower token consumption. However, failures at any point can terminate the query without extracting any information (Supplementary Section S4). Parallel workflows better scale to future throughputs with the elevated token consumption offset by an expected decrease in cost.

Overall, Gemma 2 was the most suitable open model for both parallel and multistep workflows (Supplementary Tables S4.1 and S4.2) with average F1 scores of 0.92 and 0.81 for scope, granularity, variant, and filter extraction whereas GPT models were the most suitable commercial models with F1 scores of 0.91 and 0.81, respectively. Gemma 2’s superior performance in parallel workflow may be due to its training on knowledge distillation rather than the next token prediction of GPT-4 (Riviere *et al.* 2024). Commercial models overall had greater performance than open-weight models, probably due to the larger model size and up-to-date knowledge of bioinformatics and genome beacons. We have also presented LLM specific analyses of performance in Supplementary Section S5.

### 3.3 Context management for building progressively complex interactions across multiple topics

AskBeacon manages chat histories to enable users to build on previous queries. These history objects contain the summary of a conversation, specifically the variants, filters, chosen scope, and granularity. This enables AskBeacon to request additional information to generate a Beacon compliant query. As shown in Fig. 1.3 the sBeacon User Interface (UI) allows the addition of multiple tabs so different queries can be maintained in parallel, enabling concurrent but independent querying.

### 3.4 AskBeacon keeps the human in the loop

AskBeacon keeps data extraction separate from data analysis to enable checkpointing from the human expert. All inferences are verified with the user to ensure elements such as ontology terms or genomic locations queried are aligned with the research question (Fig. 1.2). For users with programming capability the generated code for data transformation and plotting can be reviewed and amended in an editor before execution (Fig. 1.5). This optional step is further supported by also displaying both standard output and error streams aiding debugging (Supplementary Section S6).

### 3.5 Security measures

The analysis and exchange of sensitive data demands strict guard-rails to prevent misuse, leakage, or exploitation by malicious actors. While all LLM vendors assure data privacy and security contractually, AskBeacon augments this by keeping sBeacon as the conduit between the data and the LLM, so that data is never exposed to the LLM directly. sBeacon ensures the safety of underlying data through state-of-the-art data security, with user management adhering to GA4GH protocol guidelines. sBeacon also ensures that AskBeacon’s data extractors operate at the same level of access as the active user, hence no data can be extracted through AskBeacon that the user does not already have access to. AskBeacon’s analytics is protected by using static code analysis and sandboxing, preventing accidental exposure of source code, files, or runtime variables. We also perform static code analysis before any code execution and remove code with adversarial effect.

## 4 Conclusion

We built AskBeacon to leverage the GA4GH’s Beacon protocol and LLMs to enable genomic data analysis using natural language across the global Beacon network. In the future, we will extend the conversation capability to combine data extraction and analytics task, while retaining our extensive human checkpointing. This will enable users to ask comparative questions between cohorts, with AskBeacon automatically choosing the best statistical and visualization method. Given that Beacon is a discovery tool, differences in return values need to be considered, e.g. while some beacons return individual genotypes others might return summary statistics that are incompatible with each other. This also extends to differences in ontologies. We will leverage efforts from the terminology community to translate between different dictionaries and develop principles for beacons to communicate across different abstraction levels.

## Supplementary Material

btaf079_Supplementary_Data

## Data Availability

Data used in the preparation of this article were obtained on 30 September 2024 from the PPMI database (https://www.ppmi-info.org/access-data-specimens/download-data), RRID: SCR_006431. For up-to-date information on the study, visit http://www.ppmi-info.org.

## References

[btaf079-B1] Achiam J , Adler S, Agarwal S et al; OpenAI. *GPT-4 Technical Report*. 2023. https://arxiv.org/abs/2303.08774

[btaf079-B2] Angelini G , Malvaso A, Schirripa A et al Unraveling sex differences in Parkinson’s disease through explainable machine learning. J Neurol Sci 2024;462:123091. 10.1016/j.jns.2024.123091 38870732

[btaf079-B3] Anthropic AI. The Claude 3 model family: Opus, sonnet, haiku. Claude-3 Model Card 2024;1. https://www.anthropic.com/news/claude-3-5-sonnet, https://www-cdn.anthropic.com/de8ba9b01c9ab7cbabf5c33b80b7bbc618857627/Model_Card_Claude_3.pdf

[btaf079-B4] Chahine LM , Urbe L, Caspell-Garcia C et al; Parkinson’s Progression Markers Initiative. Cognition among individuals along a spectrum of increased risk for Parkinson’s disease. PLoS ONE 2018;13:e0201964. 10.1371/journal.pone.0201964 30125297 PMC6101368

[btaf079-B5] Fiume M , Cupak M, Keenan S et al Federated discovery and sharing of genomic data using Beacons. Nat Biotechnol 2019;37:220–4. 10.1038/s41587-019-0046-x 30833764 PMC6728157

[btaf079-B6] Fromont LA , Moldes M, Baudis M et al Twelve quick tips for deploying a Beacon. PLoS Comput Biol 2024;20:e1011817. 10.1371/journal.pcbi.1011817 38427629 PMC10906850

[btaf079-B7] Klein C , König IR. Exploring uncharted territory: genetically determined sex differences in Parkinson’s disease. Ann Neurol 2021;90:15–8. 10.1002/ana.26091 33938006

[btaf079-B8] Le Guen Y , Napolioni V, Belloy ME et al Common X‐chromosome variants are associated with Parkinson disease risk. Ann Neurol 2021;90:22–34. 10.1002/ana.26051 33583074 PMC8601399

[btaf079-B9] Ollama. 2024. https://github.com/ollama/ollama

[btaf079-B10] Riviere M , Pathak S, Sessa PG et al; Gemma Team. Gemma 2: improving open language models at a practical size. 2024. https://arxiv.org/abs/2408.00118

[btaf079-B11] Rueda M , Ariosa R, Moldes M et al Beacon v2 reference implementation: a toolkit to enable federated sharing of genomic and phenotypic data. Bioinformatics 2022;38:4656–7. 10.1093/bioinformatics/btac568 35980167

[btaf079-B12] Sever R. We need a plan D. Nat Methods 2023;20:473–4. 10.1038/s41592-023-01817-y 36879044

[btaf079-B13] Vaswani A , Shazeer N, Parmar N et al *Attention Is All You Need*. 2017. https://arxiv.org/abs/1706.03762

[btaf079-B14] Wickramarachchi A , Hosking B, Jain Y et al Scalable genomic data exchange and analytics with sBeacon. Nat Biotechnol 2023;41:1510–2. 10.1038/s41587-023-01972-9 37709914

[btaf079-B15] Ye J , Chen X, Xu N et al *A Comprehensive Capability Analysis of GPT-3 and GPT-3.5 Series Models*. 2023. https://arxiv.org/abs/2303.10420

